# Leukocyte dynamics in female reproductive health: roles and mechanisms

**DOI:** 10.1097/MS9.0000000000002926

**Published:** 2025-05-21

**Authors:** Emmanuel Ifeanyi Obeagu, Getrude Uzoma Obeagu

**Affiliations:** a Department of Biomedical and Laboratory Science, Africa University, Mutare, Zimbabwe; b School of Nursing Science, Kampala International University, Ishaka, Uganda

**Keywords:** female reproductive health, immune cells, leukocytes, menstrual cycle, pregnancy

## Abstract

Leukocytes are critical mediators of immune responses and play multifaceted roles in female reproductive health, influencing processes such as menstruation, ovulation, implantation, pregnancy, and parturition. This review examines the dynamic involvement of key leukocyte populations, including neutrophils, macrophages, T lymphocytes, natural killer (NK) cells, and dendritic cells, across reproductive processes. Leukocytes contribute to tissue remodeling, hormonal regulation, immune tolerance, and pathogen defense. Dysregulation in their functions is implicated in reproductive disorders such as endometriosis, recurrent pregnancy loss (RPL), and complications arising from infections. The review integrates emerging insights into the molecular mechanisms governing leukocyte behavior, emphasizing the roles of cytokines, hormones, and chemokines in guiding their recruitment and activity. Key findings underscore the significance of leukocyte-mediated cytokine networks in maintaining immune homeostasis during pregnancy and their critical roles in spiral artery remodeling for fetal-maternal exchange. Neutrophils and macrophages support menstrual shedding and repair, while NK cells facilitate trophoblast invasion and placental development. Dysregulated leukocyte activity contributes to chronic inflammation in endometriosis and impaired immune tolerance in RPL. Additionally, leukocytes are central to immune defenses against infections, but excessive inflammation can lead to infertility or adverse pregnancy outcomes. Therapeutic strategies targeting these immune cells hold promise for managing reproductive health disorders by modulating inflammation, enhancing immune tolerance, and developing biomarkers for early diagnosis. In conclusion, leukocyte dynamics are integral to reproductive physiology and pathology, with significant potential for translational research to optimize reproductive health outcomes.

## Introduction

Leukocytes, or white blood cells, are essential components of the immune system, known for their roles in defending the body against infections and facilitating immune responses^[1]^. Beyond their traditional functions, leukocytes have significant roles in various physiological processes within the female reproductive system. This review aims to explore the intricate dynamics of leukocytes in female reproductive health, highlighting their contributions to normal reproductive functions and their involvement in reproductive disorders. The female reproductive system undergoes cyclical changes driven by hormonal fluctuations, which require precise regulation by immune cells^[2]^. Leukocytes are integral to this regulation, participating in processes such as follicular development, ovulation, menstruation, and tissue remodeling. Their ability to secrete cytokines, chemokines, and growth factors allows them to interact closely with reproductive tissues, influencing cellular activities and maintaining homeostasis. During the menstrual cycle, leukocytes are actively involved in both the proliferative and secretory phases^[3]^. In the proliferative phase, macrophages and neutrophils contribute to tissue growth and repair, while in the secretory phase, they assist in preparing the endometrium for potential embryo implantation. The cyclical recruitment and activation of these immune cells are essential for maintaining the endometrial environment conducive to pregnancy.

Pregnancy introduces a unique immunological challenge, as the maternal immune system must tolerate the semi-allogeneic fetus while still protecting against infections. Uterine natural killer (uNK) cells play a pivotal role in this process by promoting the decidualization of the endometrium and supporting placental development^[4]^. Regulatory T cells (Tregs) further help in establishing immune tolerance, preventing maternal immune rejection of the fetus. Leukocytes also contribute to the maintenance of pregnancy and the initiation of labor. During pregnancy, macrophages and other immune cells are involved in remodeling the uterine tissues to accommodate the growing fetus^[5]^. As labor approaches, a coordinated inflammatory response involving leukocytes helps in cervical ripening and the eventual expulsion of the fetus. The balance between pro-inflammatory and anti-inflammatory signals is crucial for a successful pregnancy and delivery. In pathological conditions such as endometriosis, polycystic ovary syndrome (PCOS), and recurrent pregnancy loss (RPL), leukocytes often exhibit dysregulated activity^[6]^. Endometriosis involves the presence of endometrial-like tissue outside the uterus, leading to chronic inflammation driven by activated macrophages and T cells. In PCOS, chronic low-grade inflammation mediated by leukocytes contributes to metabolic and reproductive abnormalities. Recurrent pregnancy loss can be associated with abnormal activation of natural killer cells and insufficient Treg function, leading to impaired immune tolerance.

Understanding the specific mechanisms by which leukocytes contribute to these reproductive disorders is crucial for developing targeted therapies. For instance, modulating the activity of specific leukocyte subsets or their secreted factors could offer new therapeutic approaches for managing these conditions. Advances in immunological research have provided insights into the complex interactions between leukocytes and reproductive tissues, opening avenues for innovative treatments. Furthermore, the interactions between leukocytes and hormones are a critical aspect of reproductive health^[7]^. Hormonal fluctuations during the menstrual cycle and pregnancy influence leukocyte function and distribution. Estrogen and progesterone, in particular, have been shown to modulate immune cell activity, affecting their ability to respond to infections and participate in tissue remodeling. The reproductive tract’s immune environment is unique, balancing the need for immune protection with the necessity of immune tolerance^[8]^. This balance is maintained by a diverse population of leukocytes, including macrophages, neutrophils, natural killer cells, and T cells, each playing distinct roles in reproductive health. The dynamic interactions among these cells and reproductive tissues ensure the proper functioning of the reproductive system. Leukocytes are indispensable to female reproductive health, influencing various physiological processes and contributing to the pathogenesis of reproductive disorders. This review aims to provide a comprehensive overview of the roles and mechanisms of leukocytes in the female reproductive system, highlighting their importance and the potential for therapeutic interventions.

The novelty of this review lies in its detailed exploration of leukocyte heterogeneity and functionality within the reproductive system, coupled with an analysis of their molecular regulation through cytokines, chemokines, and hormonal signals. Unlike previous studies, this review also highlights the translational potential of targeting leukocyte-mediated pathways for diagnosing and treating reproductive disorders, including endometriosis, recurrent pregnancy loss, and infections. By bridging immunology and reproductive health, this review provides a novel framework to guide future research and therapeutic interventions.

## Aim

The aim of this comprehensive review is to explore the dynamic roles and mechanisms of leukocytes in female reproductive health.

## Rationale

The aim of this comprehensive review is to explore the dynamic roles and mechanisms of leukocytes in female reproductive health. By examining their contributions across various physiological processes, including the menstrual cycle, pregnancy, and reproductive disorders, this review seeks to provide insights into the intricate interactions between leukocytes and reproductive tissues. Through a thorough analysis of the mechanisms of leukocyte action, including chemotaxis, phagocytosis, cytokine production, and hormonal regulation, the aim is to deepen our understanding of their roles in maintaining tissue homeostasis, immune defense, and immune tolerance in the female reproductive system. Furthermore, this review aims to highlight the clinical implications of leukocyte dysregulation in reproductive disorders such as endometriosis, PCOS, RPL, and preeclampsia. By elucidating the specific mechanisms by which leukocytes contribute to these conditions, the aim is to identify potential therapeutic targets and strategies for improving reproductive health outcomes. Additionally, the aim is to underscore the importance of ongoing research in uncovering the complexities of leukocyte function in reproductive health and disease, with the ultimate goal of advancing reproductive medicine and enhancing patient care.

## Review methodology

### Search strategy

A systematic search strategy was developed to identify relevant literature from electronic databases, including PubMed, Scopus, and Web of Science. The search strategy employed a combination of keywords related to leukocytes, female reproductive health, and specific reproductive processes and disorders. Boolean operators and truncation were used to broaden the search and capture relevant studies.

### Inclusion and exclusion criteria

Inclusion criteria were established to ensure that studies selected for review were relevant to the topic and met predefined criteria. Studies focusing on leukocyte biology, reproductive physiology, pregnancy, and reproductive disorders in humans were considered. Both experimental and observational studies, including clinical trials, cohort studies, case-control studies, and systematic reviews, were included. Animal studies and studies not available in English were excluded.

### Study selection

Following the initial database search, duplicate records were removed using reference management software. Titles and abstracts of remaining studies were screened independently by two reviewers to assess eligibility based on the predefined inclusion criteria. Full-text articles of potentially relevant studies were then retrieved and further assessed for eligibility. Any discrepancies between reviewers were resolved through discussion and consensus.

### Data extraction

Data extraction was performed independently by two reviewers using a standardized data extraction form. Relevant information extracted from each study included study characteristics (e.g., study design, sample size), participant demographics, leukocyte populations examined, reproductive processes or disorders studied, and key findings related to leukocyte roles and mechanisms.

### Quality assessment

The quality and risk of bias of included studies were assessed using appropriate tools depending on study design. For randomized controlled trials, the Cochrane Collaboration’s Risk of Bias tool was used. For observational studies, tools such as the Newcastle-Ottawa Scale for cohort studies and the Joanna Briggs Institute Critical Appraisal Checklist for case-control studies were employed. Studies were evaluated based on criteria such as sample representativeness, study design, outcome assessment, and statistical analysis.

## Leukocytes in the female reproductive tract

The female reproductive tract is a meticulously regulated microenvironment where a symphony of cellular interactions unfolds to facilitate fertility^[9]^. While traditionally recognized for their role in immune defense, leukocytes, comprising a diverse array of immune cells, are gaining prominence for their nuanced contributions within this reproductive milieu. This exploration aims to unravel the presence, types, and potential functions of leukocytes in the female reproductive tract, shedding light on their dynamic roles beyond conventional immune functions. Leukocytes, including neutrophils, macrophages, and lymphocytes, are not merely bystanders but active participants in the female reproductive tract^[10]^. This section delves into the distribution of leukocytes across key reproductive tissues, exploring their abundance in the uterus, fallopian tubes, cervix, and other anatomical sites.^[11]^ Leukocytes contribute significantly to this complex milieu, engaging in interactions with other immune cells, epithelial cells, and resident microbes. This section elucidates how leukocytes shape the local immune landscape, fostering an environment that supports reproductive events.

Beyond their conventional roles as defenders against pathogens, leukocytes engage in immune surveillance and maintenance of homeostasis within the female reproductive tract. This section explores how leukocytes act as sentinels, patrolling for potential threats while simultaneously contributing to tissue repair and remodeling. The delicate balance between immune vigilance and tissue homeostasis is central to understanding the multifaceted functions of leukocytes. The composition and activity of leukocytes in the female reproductive tract undergo dynamic changes across the menstrual cycle^[12]^. Hormonal fluctuations influence the immune landscape, impacting the abundance and functions of leukocytes. This section delves into the temporal variations in leukocyte populations, emphasizing their responsiveness to hormonal cues and their potential roles in orchestrating cyclic reproductive events. The cervical mucus, a crucial player in sperm transport and protection, is intricately linked with leukocyte activity^[13]^. This section explores the interactions between leukocytes and cervical mucus, investigating how these immune cells may contribute to the modulation of mucus properties and, consequently, fertility. This exploration highlights the active and dynamic presence of leukocytes in the female reproductive tract. Beyond their traditional immune functions, leukocytes contribute to the orchestration of reproductive events, from immune surveillance and tissue homeostasis to their involvement in cyclic processes across the menstrual cycle.

### Leukocytes and the menstrual cycle

The menstrual cycle is a complex, hormonally-driven process that prepares the female reproductive system for potential pregnancy^[14]^. It consists of four main phases: the menstrual phase, the follicular phase, ovulation, and the luteal phase. Each phase is characterized by distinct hormonal changes and physiological events, which are tightly regulated by the endocrine system. Leukocytes play crucial roles throughout the menstrual cycle, contributing to tissue remodeling, immune defense, and the regulation of inflammatory processes.

#### Leukocytes in the menstrual phase

During the menstrual phase, the functional layer of the endometrium is shed, resulting in menstrual bleeding. Leukocytes, particularly neutrophils and macrophages, are heavily involved in this process. These cells infiltrate the endometrium and secrete enzymes and cytokines that help break down the tissue. Neutrophils release proteolytic enzymes such as matrix metalloproteinases (MMPs) that degrade the extracellular matrix, facilitating the shedding of the endometrial lining^[15]^. Macrophages, on the other hand, phagocytose cellular debris and apoptotic cells, aiding in tissue clearance and preventing infections.

#### Leukocytes in the follicular phase

The follicular phase is marked by the proliferation and regeneration of the endometrial lining, driven by rising estrogen levels. During this phase, macrophages and other immune cells contribute to tissue repair and regeneration. Macrophages secrete growth factors and cytokines that promote the proliferation of endometrial stromal and epithelial cells^[16]^. Additionally, macrophages play a role in angiogenesis, the formation of new blood vessels, which is essential for providing nutrients and oxygen to the regenerating endometrial tissue.

#### Leukocytes and ovulation

Ovulation is the release of an egg from the ovarian follicle, triggered by a surge in luteinizing hormone (LH). This process is accompanied by localized inflammation and the involvement of immune cells. Leukocytes, including macrophages and neutrophils, accumulate in the ovary around the time of ovulation^[17]^. These cells produce inflammatory mediators and enzymes that help break down the follicular wall, facilitating the release of the oocyte. The presence of leukocytes ensures the timely rupture of the follicle and the successful release of the egg.

#### Leukocytes in the luteal phase

The luteal phase follows ovulation and is characterized by the formation of the corpus luteum, which secretes progesterone to support potential pregnancy. Leukocytes continue to play roles in maintaining the endometrium during this phase^[18]^. Macrophages and other immune cells help modulate the immune environment to favor implantation and early pregnancy. They secrete cytokines that support the stability and receptivity of the endometrial lining, creating an optimal environment for embryo implantation.

#### Immune tolerance and the menstrual cycle

Throughout the menstrual cycle, leukocytes help maintain a balance between immune tolerance and defense^[12]^. The endometrial lining must be protected from infections while also being receptive to potential embryo implantation. Tregs are particularly important in this regard, as they help suppress excessive immune responses that could harm the endometrium or interfere with implantation. The cyclical recruitment and activation of leukocytes ensure that the endometrium remains both protected and receptive.

#### Leukocyte recruitment and chemotaxis

The recruitment of leukocytes to the endometrium is a tightly regulated process involving chemotactic signals. Chemokines such as CCL2 and CXCL12 play crucial roles in attracting leukocytes to the endometrial tissue^[19]^. Estrogen and progesterone modulate the expression of these chemokines, influencing leukocyte migration and activity. This hormonal regulation ensures that leukocytes are present in the endometrium at appropriate times to carry out their functions.

#### Hormonal regulation of leukocyte activity

Hormones such as estrogen and progesterone not only influence leukocyte recruitment but also modulate their activity^[20]^. Estrogen has been shown to enhance the phagocytic activity of macrophages and the production of anti-inflammatory cytokines. Progesterone, on the other hand, promotes an anti-inflammatory environment by increasing the production of Tregs and reducing the activity of pro-inflammatory cells. This hormonal regulation ensures that leukocytes contribute to tissue homeostasis and repair without causing excessive inflammation.

#### Clinical implications of leukocyte dysfunction

Dysfunction in leukocyte activity can lead to menstrual disorders and reproductive health issues^[7]^. For example, excessive inflammation due to overactive leukocytes can contribute to conditions such as dysmenorrhea (painful menstruation) and menorrhagia (heavy menstrual bleeding). Conversely, insufficient leukocyte activity can impair tissue repair and increase susceptibility to infections. Understanding the roles of leukocytes in the menstrual cycle can inform the development of treatments for these conditions.

### Leukocytes in pregnancy

Pregnancy is a unique immunological state where the maternal immune system must adapt to accommodate and support the semi-allogeneic fetus. This adaptation involves a complex interplay between various leukocyte populations and immune regulatory mechanisms. The successful progression of pregnancy relies on leukocytes to facilitate implantation, establish immune tolerance, and support fetal development while protecting against infections.^[^21-23^]^ The implantation of the embryo into the maternal endometrium is a critical step in early pregnancy, requiring a finely tuned immune response. uNK cells are the most abundant leukocytes in the decidua (the modified endometrial lining during pregnancy)^[24]^. uNK cells play a crucial role in remodeling maternal spiral arteries to ensure adequate blood flow to the developing placenta. They secrete angiogenic factors and cytokines, such as VEGF (vascular endothelial growth factor) and IFN-γ (interferon-gamma), which promote vascular changes and support trophoblast invasion. One of the most remarkable aspects of pregnancy is the establishment of immune tolerance to the fetus. Tregs are essential for maintaining this tolerance. Tregs suppress maternal immune responses that could target fetal antigens, thus preventing fetal rejection. The expansion and activity of Tregs are enhanced during pregnancy, facilitated by pregnancy hormones such as progesterone and human chorionic gonadotropin (hCG). These hormones promote the expression of immunosuppressive cytokines like IL-10 and TGF-β, further supporting immune tolerance. Leukocytes are integral to placental development and function. Macrophages, another prominent leukocyte population in the decidua, contribute to placental development by clearing apoptotic cells and participating in tissue remodeling. They produce growth factors and cytokines that support the growth and differentiation of trophoblasts, the cells that form the outer layer of the placenta. Decidual macrophages also help maintain a balance between pro-inflammatory and anti-inflammatory responses, crucial for a healthy pregnancy^[24]^.

Throughout mid to late pregnancy, leukocytes continue to play supportive roles. They are involved in modulating the maternal immune system to prevent excessive inflammation that could harm the fetus. For instance, decidual macrophages and Tregs work together to maintain a state of controlled immune activation, protecting the maternal-fetal interface from infections while preventing adverse immune reactions. This balance is essential for fetal growth and development. As pregnancy progresses toward term, leukocytes contribute to the preparation for labor. Neutrophils and macrophages infiltrate the cervix and myometrium, where they produce cytokines and enzymes that promote cervical ripening and uterine contractions^[25]^. The inflammatory milieu created by these leukocytes is necessary for the initiation of labor. For example, macrophages release prostaglandins and MMPs, which help break down cervical collagen, facilitating cervical dilation and effacement. During labor, the immune environment shifts to facilitate the delivery process. An influx of leukocytes, particularly neutrophils, into the maternal-fetal interface enhances the inflammatory response needed for labor. This response is tightly regulated to ensure effective labor while minimizing potential tissue damage. The release of pro-inflammatory cytokines, such as IL-1 and TNF-α, by leukocytes drives the inflammatory cascade that promotes uterine contractions and the expulsion of the fetus.

After delivery, leukocytes are involved in postpartum recovery and tissue repair^[26]^. Macrophages play a significant role in the resolution of inflammation and the repair of the endometrial lining. They clear residual placental tissue and apoptotic cells, promoting the restoration of normal uterine function. Additionally, the immune system gradually returns to its non-pregnant state, with leukocytes helping to re-establish immune homeostasis. Dysregulation of leukocyte function can lead to pregnancy complications. For example, inadequate uNK cell activity or abnormal Treg function can contribute to pregnancy disorders such as preeclampsia and recurrent pregnancy loss. Preeclampsia is characterized by defective placental development and systemic inflammation, partly driven by an imbalance in leukocyte populations and their cytokine profiles. Similarly, insufficient immune tolerance mechanisms can lead to immune-mediated pregnancy loss. Modulating leukocyte activity through targeted therapies could help manage pregnancy complications^[5]^. For instance, enhancing Treg function or correcting uNK cell deficiencies could improve outcomes in recurrent pregnancy loss and preeclampsia. Future research should focus on identifying specific leukocyte subsets and their molecular pathways involved in pregnancy maintenance and complications. Advancements in immunotherapy and precision medicine offer promising avenues for improving maternal and fetal health.

## Immunomodulation and pregnancy

Pregnancy represents a remarkable physiological state wherein the maternal immune system undergoes dynamic adaptations to accommodate and nurture the developing fetus. Immunomodulation, the intricate regulation of immune responses, plays a central role in orchestrating the delicate balance between tolerance to the semi-allogeneic fetus and the maintenance of protective immunity^[^27,28^]^. Early pregnancy initiates a cascade of immune adaptations aimed at creating an environment conducive to embryonic implantation and fetal development.^[^29,30^]^ As trophoblast cells invade the maternal decidua, immune tolerance becomes paramount for the survival of the semi-allogeneic fetus^[31]^. Regulatory T cells emerge as pivotal orchestrators of immune tolerance during pregnancy. The cytokine milieu within the maternal-fetal interface undergoes dynamic shifts to support pregnancy. Placental development and function are intricately linked with immune modulation. Complications such as preeclampsia and recurrent pregnancy loss underscore the delicate nature of immunomodulation during pregnancy. As our understanding of immunomodulation during pregnancy advances, so do opportunities for therapeutic interventions. The intricate dance of immunomodulation during pregnancy orchestrates a harmonious relationship between the maternal immune system and the developing fetus.

## Inflammatory responses and reproductive outcomes

Inflammation, a fundamental aspect of the immune response, plays a dual role in the realm of reproductive health, influencing crucial processes such as oocyte quality, embryo development, and the establishment of a receptive endometrial environment^[32]^. The quality of oocytes, essential for successful fertilization and embryonic development, is intricately linked to inflammatory processes. The process of implantation represents a critical phase in early pregnancy, demanding precise coordination between the developing embryo and the receptive endometrium. A dynamic interplay between the immune system and the endometrium is essential for establishing an environment supportive of embryo implantation. Inflammation can exert both positive and negative influences on embryonic development. While acute inflammation is a physiological response, chronic inflammation can have detrimental effects on reproductive health. Recurrent pregnancy loss remains a complex challenge in reproductive medicine. Recognizing the pivotal role of inflammation in reproductive outcomes opens avenues for therapeutic interventions. The intricate relationship between inflammatory responses and reproductive outcomes underscores the multifaceted nature of immune modulation in the context of fertility.

## Leukocytes and ovulation

Ovulation, a pivotal event in the female reproductive cycle, involves a coordinated interplay of hormonal fluctuations, ovarian processes, and intricate interactions within the follicular microenvironment. Recently, attention has turned to the potential influence of leukocytes on the ovulatory process, adding a novel dimension to our understanding of this intricate physiological phenomenon^[33]^. The presence of leukocytes within the follicular microenvironment challenges traditional views of the ovarian milieu as an immune-privileged site. Evidence suggests that leukocytes may exert immunomodulatory functions during the ovulatory phase. The cumulus-oocyte complex represents a critical nexus in the ovulatory process, facilitating oocyte maturation and subsequent fertilization. Inflammation, typically associated with immune responses, may play a nuanced role in ovulation. Dynamic changes in leukocyte populations occur during the periovulatory phase, suggesting their active participation in the events leading up to and immediately following ovulation. Dysregulation in leukocyte function may have implications for ovulatory processes and subsequent fertility outcomes. Recognizing the potential role of leukocytes in ovulation opens avenues for therapeutic exploration. The evolving understanding of leukocyte involvement in ovulation adds a layer of complexity to our comprehension of reproductive physiology.

## Leukocytes in reproductive disorders

### Endometriosis

Endometriosis is a chronic inflammatory condition characterized by the presence of endometrial-like tissue outside the uterus, leading to pain and infertility. Leukocytes, especially macrophages and T cells, play a pivotal role in the pathogenesis of endometriosis^[34]^. The ectopic endometrial tissue recruits macrophages, which produce pro-inflammatory cytokines such as TNF-α, IL-1β, and IL-6. These cytokines perpetuate inflammation, promote angiogenesis, and support the survival of ectopic lesions. Additionally, T cells in endometriotic lesions exhibit altered immune responses, contributing to a chronic inflammatory state and further exacerbating the condition.

### Polycystic ovary syndrome (PCOS)

Polycystic ovary syndrome (PCOS) is a common endocrine disorder characterized by hyperandrogenism, ovulatory dysfunction, and polycystic ovaries. Chronic low-grade inflammation is a hallmark of PCOS, with leukocytes playing a central role. Elevated levels of pro-inflammatory cytokines, such as IL-6, TNF-α, and CRP, have been observed in women with PCOS^[35]^. Macrophages and other immune cells infiltrate ovarian tissues, contributing to insulin resistance and hyperandrogenism. The chronic inflammatory state in PCOS not only affects metabolic processes but also impairs follicular development and ovulation, leading to fertility issues.

### Recurrent pregnancy loss (RPL)

Recurrent pregnancy loss (RPL) is defined as the loss of two or more consecutive pregnancies. Immune dysregulation, including abnormal leukocyte activity, is a significant factor in RPL. Natural killer (NK) cells, particularly uNK cells, are crucial for early pregnancy success^[35]^. However, aberrant activation or high numbers of peripheral NK cells have been associated with RPL. Additionally, insufficient function of Tregs can lead to inadequate immune tolerance of the fetus, resulting in pregnancy loss. Identifying and correcting these immune imbalances are key to improving outcomes in women with RPL.

### Preeclampsia

Preeclampsia is a pregnancy complication characterized by high blood pressure and damage to organ systems, often involving the kidneys. The condition is linked to defective placental development and an imbalance in immune responses. Leukocytes, particularly macrophages and T cells, contribute to the inflammatory milieu seen in preeclampsia^[36]^. Excessive activation of these immune cells leads to increased production of pro-inflammatory cytokines, oxidative stress, and endothelial dysfunction. Understanding the role of leukocytes in preeclampsia can aid in developing therapeutic interventions to modulate immune responses and improve placental function.

### Implantation failure

Implantation failure, a significant cause of infertility, occurs when the embryo fails to attach to the endometrial lining. Leukocytes, including uNK cells and macrophages, are critical for creating a receptive endometrial environment^[37]^. Dysregulation in the number or function of these immune cells can lead to implantation failure. For instance, inadequate uNK cell activity may result in insufficient remodeling of maternal spiral arteries, leading to poor placental development. Additionally, an imbalance between pro-inflammatory and anti-inflammatory cytokines can disrupt the endometrial receptivity, hindering embryo implantation.

### Uterine fibroids

Uterine fibroids are benign tumors of the uterus that can cause heavy menstrual bleeding, pain, and reproductive issues. Leukocytes, particularly macrophages, are implicated in the pathogenesis of fibroids^[38]^. Infiltration of macrophages into fibroid tissues leads to the production of growth factors and cytokines that promote fibroid growth and extracellular matrix production. The inflammatory environment created by these immune cells can exacerbate fibroid symptoms and contribute to their persistence and enlargement.

### Pelvic inflammatory disease (PID)

Pelvic inflammatory disease (PID) is an infection of the female reproductive organs, often caused by sexually transmitted bacteria. Leukocytes, especially neutrophils and macrophages, are the first line of defense against infections in PID^[39]^. These immune cells infiltrate the infected tissues, phagocytose bacteria, and release antimicrobial peptides and inflammatory cytokines. While their activity is crucial for combating infection, excessive inflammation can lead to scarring and damage to the reproductive organs, resulting in chronic pain, infertility, and ectopic pregnancy.

### Autoimmune disorders

Autoimmune disorders, such as lupus and antiphospholipid syndrome (APS), can significantly impact female reproductive health^[40]^. In these conditions, leukocytes mistakenly attack the body’s own tissues, including reproductive organs. For instance, in APS, autoantibodies target phospholipids in cell membranes, leading to blood clot formation and pregnancy complications such as recurrent miscarriage and preeclampsia. Managing autoimmune disorders often involves immunosuppressive therapies to reduce leukocyte-mediated damage and improve reproductive outcomes.

### Chronic endometritis

Chronic endometritis is a persistent inflammation of the endometrial lining often associated with infertility and recurrent implantation failure. The condition is characterized by the presence of plasma cells and other leukocytes in the endometrium. These immune cells produce inflammatory cytokines and chemokines that disrupt the normal endometrial environment, making it inhospitable for embryo implantation. Antibiotic treatment targeting the underlying infection can reduce inflammation and improve fertility outcomes in affected women.

## Mechanisms of leukocyte action

### Chemotaxis and recruitment

Chemotaxis is the process by which leukocytes are guided to sites of infection, inflammation, or tissue remodeling in response to chemical signals^[41]^. In the reproductive system, chemotactic signals such as chemokines and cytokines are crucial for directing leukocytes to specific locations where they are needed. For instance, during the menstrual cycle, chemokines like CCL2 and CXCL12 are upregulated to attract macrophages and neutrophils to the endometrium, facilitating tissue repair and regeneration. Similarly, during pregnancy, the decidua secretes chemokines that recruit uNK cells and macrophages to support placental development and immune tolerance.

### Phagocytosis and clearance

Phagocytosis is a key mechanism by which leukocytes, particularly macrophages and neutrophils, engulf and digest cellular debris, pathogens, and apoptotic cells^[42]^. In reproductive health, phagocytosis is essential for maintaining tissue homeostasis. During menstruation, macrophages clear apoptotic endometrial cells and tissue fragments, preventing infection and promoting tissue renewal. In pregnancy, decidual macrophages phagocytose apoptotic trophoblasts and other cellular debris, ensuring a clean environment for the developing fetus and preventing excessive inflammation.

### Cytokine production

Leukocytes produce a wide array of cytokines that regulate immune responses, tissue remodeling, and cellular communication^[43]^. These cytokines can be pro-inflammatory, promoting inflammation and immune activation, or anti-inflammatory, suppressing immune responses and facilitating tissue repair. For example, during the menstrual cycle, macrophages produce pro-inflammatory cytokines like TNF-α and IL-1β to aid in endometrial shedding, followed by anti-inflammatory cytokines such as IL-10 to promote tissue repair. In pregnancy, the balance of cytokines is crucial for maintaining immune tolerance; uNK cells and Tregs secrete cytokines like IL-10 and TGF-β to support placental development and prevent maternal immune rejection of the fetus.

### Interaction with hormones

Leukocyte function in the reproductive system is closely regulated by hormonal signals, particularly estrogen and progesterone^[44]^. These hormones influence leukocyte behavior by modulating cytokine production, chemokine expression, and cell surface receptor availability. Estrogen, for instance, enhances the phagocytic activity of macrophages and promotes the production of anti-inflammatory cytokines. Progesterone increases the number and activity of Tregs and uNK cells, supporting immune tolerance and placental development. The cyclical fluctuations of these hormones throughout the menstrual cycle and pregnancy ensure that leukocytes perform their roles at the appropriate times.

### Immune modulation and tolerance

Tregs are essential for maintaining immune tolerance, especially during pregnancy^[45]^. Tregs suppress excessive immune responses that could target the semi-allogeneic fetus. They produce immunosuppressive cytokines such as IL-10 and TGF-β and interact with other immune cells to inhibit their activation. In reproductive health, Tregs are critical for preventing conditions like recurrent pregnancy loss and preeclampsia, which can result from immune intolerance and excessive inflammation. The expansion and activity of Tregs are influenced by pregnancy hormones, ensuring a conducive environment for fetal development.

### Angiogenesis and tissue remodeling

Leukocytes contribute to angiogenesis and tissue remodeling by secreting growth factors and enzymes^[46]^. Macrophages and uNK cells produce vascular endothelial growth factor (VEGF) and other angiogenic factors that promote the formation of new blood vessels, crucial for endometrial regeneration and placental development. MMPs secreted by macrophages degrade extracellular matrix components, facilitating tissue remodeling and the invasion of trophoblasts during implantation. These processes are vital for establishing a functional placenta and maintaining the health of the endometrial lining.

### Oxidative stress and antioxidant defense

Leukocytes can generate reactive oxygen species (ROS) as part of the immune response to pathogens and tissue damage^[47]^. However, excessive ROS production can lead to oxidative stress, which is implicated in reproductive disorders such as endometriosis and preeclampsia. To counteract this, leukocytes also produce antioxidant enzymes and molecules that neutralize ROS and protect tissues from oxidative damage. The balance between ROS production and antioxidant defense is crucial for maintaining reproductive health and preventing pathological conditions.

### Cell-cell communication

Leukocytes communicate with other cells in the reproductive system through direct cell-cell contact and the release of soluble mediators. Cell surface receptors such as integrins and selectins facilitate the adhesion and migration of leukocytes to target tissues. Additionally, leukocytes release extracellular vesicles (EVs) that carry signaling molecules, including cytokines, chemokines, and microRNAs, which influence the behavior of surrounding cells^[48]^. This intricate communication network ensures coordinated immune responses and tissue remodeling processes essential for reproductive health.

### Epigenetic regulation

Leukocyte function is also modulated by epigenetic mechanisms, which involve changes in gene expression without altering the DNA sequence. Epigenetic modifications such as DNA methylation, histone acetylation, and non-coding RNA regulation can influence leukocyte activity, differentiation, and cytokine production^[49]^. In the reproductive system, hormonal changes can induce epigenetic alterations in leukocytes, affecting their responses during the menstrual cycle and pregnancy. Understanding these epigenetic mechanisms can provide insights into the regulation of leukocyte function and the development of reproductive disorders.

## Chronic inflammation and infertility

Chronic inflammation can potentially have an impact on fertility, although the relationship is complex and may involve various factors. Inflammation is part of the body’s natural immune response to injury or infection. However, when inflammation becomes chronic, it can lead to a range of health issues. Chronic inflammation can damage reproductive organs, such as the ovaries, fallopian tubes, and uterus^[50]^. This damage may interfere with normal reproductive processes, including ovulation, fertilization, and implantation. Inflammation can disrupt the delicate balance of hormones involved in the menstrual cycle and reproduction. Hormonal imbalances can affect the regularity of menstrual cycles, ovulation, and the overall fertility of both men and women. Chronic inflammation can lead to an overactive immune response, which may negatively impact fertility. In some cases, the immune system may mistakenly target sperm or embryos, leading to difficulties in conception or early pregnancy loss. Inflammation may affect the quality of eggs and sperm. Poor egg quality or impaired sperm function can reduce the chances of successful fertilization and pregnancy. PCOS is a common condition characterized by hormonal imbalances and the development of cysts on the ovaries. Inflammation may contribute to the development and progression of PCOS, which can affect fertility. Endometriosis is a condition where tissue similar to the lining of the uterus grows outside the uterus. Inflammation is thought to play a role in the development of endometriosis, and this condition can be associated with infertility.

It’s important to note that while chronic inflammation may be a factor in infertility, it is often just one piece of a complex puzzle. Other factors such as age, genetics, lifestyle factors, and underlying medical conditions can also contribute to fertility issues. If someone is experiencing fertility problems, it’s advisable to consult with a healthcare professional or a fertility specialist. They can conduct a thorough evaluation, including medical history, physical examination, and diagnostic tests, to identify any underlying causes of infertility and develop an appropriate treatment plan. Lifestyle modifications, anti-inflammatory medications, and other interventions may be considered based on the specific circumstances.

## Diagnostic significance and biomarkers of leukocytes in women with infertility

Leukocytes, commonly known as white blood cells, are an essential part of the immune system and play a role in protecting the body against infections^[51]^. In the context of infertility, elevated levels of leukocytes or specific changes in their activity may indicate underlying issues that could impact fertility^[52]^. Elevated levels of leukocytes in the reproductive tract, particularly in the cervix or uterus, may indicate the presence of infection or inflammation. Infections, such as pelvic inflammatory disease (PID), can lead to scarring and damage to reproductive organs, potentially causing infertility. Abnormalities in the immune system, leading to an overactive or underactive response, may contribute to infertility. Leukocytes are key components of the immune system, and their dysregulation can affect reproductive processes. In some cases, an immune response against sperm or eggs may occur, preventing successful fertilization. Leukocytes can be involved in immune reactions that affect the interaction between sperm and eggs.

### Biomarkers

1. Cytokines and chemokines: These are signaling molecules released by leukocytes and other cells involved in the immune response. Elevated levels of certain cytokines or chemokines in the reproductive tract may indicate inflammation or an immune response that could impact fertility.
2. White blood cell counts: A simple blood test can measure the overall white blood cell count, providing an indication of the body’s immune response. Elevated white blood cell counts may suggest systemic inflammation or infection.
3. Presence of inflammatory mediators: Detection of specific inflammatory mediators, such as prostaglandins or interleukins, in the reproductive fluids may indicate localized inflammation that could affect fertility.
4. Immunoglobulin levels: Immunoglobulins, including IgA, IgM, and IgG, are antibodies produced by the immune system. Abnormal levels of these antibodies may suggest an immune response that could impact fertility.
5. Leukocyte esterase test: This test can be performed on vaginal or cervical fluid to detect the presence of leukocytes, providing an indication of local inflammation or infection.

### Clinical considerations

Elevated levels of leukocytes in the endometrial lining may be associated with chronic endometritis, a condition characterized by persistent inflammation of the uterine lining^[53]^. Specialized immunological tests may be conducted to assess the immune system’s response to reproductive tissues, helping to identify potential immune-related causes of infertility. It’s important to note that while leukocyte levels and immune factors can be relevant in the context of infertility, comprehensive fertility evaluations often involve a combination of tests, including hormonal assessments, imaging studies, and a thorough medical history. Consulting with a reproductive endocrinologist or fertility specialist is crucial for a personalized and thorough assessment of infertility issues, including the role of leukocytes and immune factors.

## Therapeutic implications of leukocytes in women with infertility

The therapeutic implications of leukocytes in women with infertility often depend on the specific underlying causes identified through diagnostic assessments^[54]^. Addressing leukocyte-related factors may involve a combination of medical interventions, lifestyle modifications, and targeted treatments. If infections are identified, such as those causing pelvic inflammatory disease (PID), appropriate antibiotic therapy is crucial to eliminate the infection. Anti-inflammatory medications may be considered to address inflammation in the reproductive tract. In cases where an overactive immune response is suspected, immunomodulatory therapies may be considered to regulate the immune system. Immunotherapy approaches might involve medications to suppress or modulate specific immune responses that could be interfering with fertility.^[^55-59^]^

Addressing hormonal imbalances, if identified, is essential for restoring normal reproductive function. Hormonal therapies, such as those targeting irregular menstrual cycles or ovulatory dysfunction, may be recommended^[55]^. Lifestyle factors, such as stress, smoking, and excessive alcohol consumption, can impact the immune system and fertility. Lifestyle modifications may be advised to optimize overall health and reproductive function. Addressing underlying conditions contributing to immune dysregulation or inflammation, such as endometriosis or polycystic ovary syndrome (PCOS), is important. Surgical interventions or other targeted treatments may be recommended based on the specific diagnosis. In some cases, when other treatments are not successful, assisted reproductive technologies like in vitro fertilization (IVF) may be considered. IVF can help bypass certain fertility barriers and enhance the chances of successful conception. For cases involving chronic endometritis or suspected immune-related issues impacting endometrial receptivity, specialized tests to assess endometrial receptivity may be performed.^[^60-64^]^ Tailored treatments, such as endometrial scratching or specific hormonal interventions, may be considered to improve endometrial receptivity. Collaboration with immunologists or reproductive endocrinologists with expertise in immune-related fertility issues may be beneficial in developing targeted therapeutic strategies. Regular monitoring of treatment effectiveness through diagnostic tests and imaging studies is essential. Treatment plans may need adjustments based on the individual’s response and evolving circumstances. It’s crucial for women experiencing infertility associated with leukocyte-related factors to consult with a reproductive endocrinologist or fertility specialist. A comprehensive evaluation will guide the development of an individualized treatment plan based on the specific underlying causes identified through diagnostic testing (Table 1). The goal is to address the identified factors affecting fertility and optimize the chances of a successful pregnancy.^[^65-67^]^

**Table 1:** Summary of leukocyte roles, mechanisms, and implications in female reproductive health

Leukocyte type	Key roles	Mechanisms	Implications/pathologies
Neutrophils	- Tissue breakdown during menstruation	- Release of matrix metalloproteinases (MMPs)	- Dysregulated activity linked to chronic inflammation
	- Defense against pathogens	- Secretion of pro-inflammatory cytokines	- Role in infection clearance
Macrophages	- Tissue repair	- Phagocytosis	- Aberrant activity in endometriosis
	- Regulation of ovulation and corpus luteum function	- Angiogenesis via VEGF secretion	- Impaired placental development in pregnancy complications
	- Placental development	- Modulation of inflammatory responses	
T Lymphocytes	- Immune surveillance	- Regulation by cytokines like IL-10 and TGF-β	- Insufficient tolerance linked to recurrent pregnancy loss (RPL)
	- Maintenance of immune tolerance	- Suppression by regulatory T cells (Tregs)	
NK cells	- Trophoblast invasion	- Release of angiogenic factors	- Dysregulation associated with preeclampsia and implantation failure
	- Spiral artery remodeling	- Regulation by hormonal signals	
	- Cytokine production		
Dendritic cells	- Antigen presentation	- Interaction with trophoblasts	- Disruption linked to pregnancy complications and infections
	- Modulation of maternal-fetal immune balance	- Production of immune-tolerant cytokines	
Cytokine networks	- Regulation of immune responses	- Interplay of pro- and anti-inflammatory cytokines (e.g., IFN-γ, IL-10)	- Imbalances contribute to conditions like endometriosis and preterm labor
	- Balancing inflammation and tolerance		
Hormonal modulation	- Coordination of immune responses across reproductive phases	- Estrogen and progesterone modulation of leukocyte activity	- Hormonal dysregulation exacerbates immune-related pathologies
Chemokines	- Recruitment of leukocytes to the reproductive tract	- Activation of chemokine receptors (e.g., CXCL12, CCR5)	- Dysregulated trafficking affects implantation and immune defenses

### Implications

Understanding leukocyte dynamics in female reproductive health has significant implications for both basic and applied sciences. Insights into the roles of leukocytes in immune tolerance, tissue remodeling, and pathogen defense help elucidate mechanisms underlying normal reproductive physiology and the etiology of disorders like endometriosis and recurrent pregnancy loss. Such knowledge lays the foundation for developing targeted therapeutic interventions that address immune dysregulation, reduce inflammation, and promote tissue repair in reproductive pathologies.

### Application

The findings discussed in this review have broad applicability in clinical and research contexts. They are relevant to the development of diagnostic biomarkers for reproductive health disorders, enabling early detection and intervention. Moreover, the principles of leukocyte-mediated immune modulation can be generalized to other immunologically sensitive systems, providing a template for managing conditions that involve immune-tissue interactions, such as organ transplantation or autoimmune diseases.

### Transferability

The concepts outlined in this review, such as the regulation of leukocyte activity by cytokines and hormones, can be transferred to various contexts beyond reproductive health. For example, the mechanisms of immune tolerance and inflammation explored here have parallels in cancer immunology and chronic inflammatory conditions. Furthermore, the strategies for modulating immune responses in reproductive settings may inform therapeutic approaches for systemic diseases involving leukocyte dysfunction.

### Contribution to the field

This review contributes to the field by offering a novel, integrative perspective on leukocyte dynamics within the female reproductive system, emphasizing their roles in both physiological and pathological states. It bridges gaps in current knowledge by combining molecular insights with clinical observations, providing a comprehensive framework for understanding immune-reproductive interactions. By highlighting translational opportunities, this review fosters innovation in diagnostics, therapeutics, and personalized medicine, advancing the field of reproductive immunology.

## Conclusion

Leukocytes are indispensable players in female reproductive health, orchestrating processes from menstruation to parturition through their roles in immune defense, tissue remodeling, and hormonal regulation. This review highlights the intricate dynamics of leukocyte populations, including neutrophils, macrophages, NK cells, T lymphocytes, and dendritic cells, emphasizing their contributions to normal physiology and their involvement in pathologies such as endometriosis and recurrent pregnancy loss.

## Data Availability

Not applicable.
